# An evaluation of adhesive sample holders for advanced crystallographic experiments

**DOI:** 10.1107/S1399004714014370

**Published:** 2014-08-29

**Authors:** Marco Mazzorana, Juan Sanchez-Weatherby, James Sandy, Carina M. C. Lobley, Thomas Sorensen

**Affiliations:** a Diamond Light Source, Diamond House, Harwell Science and Innovation Campus, Didcot, Oxfordshire OX11 0DE, England; b Research Complex at Harwell (RCaH), Rutherford Appleton Laboratory, Didcot, Oxfordshire OX11 0FA, England

**Keywords:** macromolecular crystallography, humidity control, Crystal Catcher, resolution, crystal packing

## Abstract

The hydration state of macromolecular crystals often affects their overall order and, ultimately, the quality of the X-ray diffraction pattern that they produce. Post-crystallization techniques that alter the solvent content of a crystal may induce rearrangement within the three-dimensional array making up the crystal, possibly resulting in more ordered packing. The hydration state of a crystal can be manipulated by exposing it to a stream of air at controlled relative humidity in which the crystal can equilibrate. This approach provides a way of exploring crystal hydration space to assess the diffraction capabilities of existing crystals. A key requirement of these experiments is to expose the crystal directly to the dehydrating environment by having the minimum amount of residual mother liquor around it. This is usually achieved by placing the crystal on a flat porous support (Kapton mesh) and removing excess liquid by wicking. Here, an alternative approach is considered whereby crystals are harvested using adhesives that capture naked crystals directly from their crystallization drop, reducing the process to a one-step procedure. The impact of using adhesives to ease the harvesting of different types of crystals is presented together with their contribution to background scattering and their usefulness in dehydration experiments. It is concluded that adhesive supports represent a valuable tool for mounting macromolecular crystals to be used in humidity-controlled experiments and to improve signal-to-noise ratios in diffraction experiments, and how they can protect crystals from modifications in the sample environment is discussed.

## Abstract 2

Commercially available adhesives have been evaluated for crystal mounting when undertaking complex macromolecular crystallography experiments. Here, their use as tools for advanced sample mounting and cryoprotection is assessed and their suitability for room-temperature data-collection and humidity-controlled studies is investigated.

## 1. Introduction   

Biological macromolecules are purified in aqueous solutions containing buffer and other chemical species. Under suitable conditions these macromolecules can pack together to form a crystalline array. These crystals still contain a large amount of solvent (between 30 and 70%) both in solvation spheres around each macromolecule and in large solvent channels (Matthews, 1968 ▶). This high solvent content means that enzymes often retain their activity within crystals (Hare *et al.*, 2012 ▶) and that ligands and other compounds can diffuse into the crystal lattice (Mozzarelli & Rossi, 1996 ▶). While this corroborates the idea that the majority of crystallographic structures obtained correspond to a natural conformation of the macromolecule, the high solvent content may lead to imperfect packing of macromolecules in the crystal, reducing the quality of the X-ray diffraction pattern. When the diffraction quality of crystals is insufficient to obtain the required data one can explore protein space (*e.g.* subdomains, mutants and shortened/lengthened/insertion variations), chemical modifications (*e.g.* lysine methylation) or look for alternative conditions in the crystallization space with the intention of finding crystals with improved diffraction properties. However, these approaches can all be time-consuming and are not infallible; therefore, it is often desirable to explore whether the poorly diffracting crystals obtained might be treated to improve the crystalline order and hence the quality of the diffraction obtained (Esnouf *et al.*, 1998 ▶; Heras & Martin, 2005 ▶).

Dehydration is one way of exploring the further diffraction potential of a crystal and can lead to dramatic improvement of the crystal diffraction quality (Russo Krauss *et al.*, 2012 ▶). In some cases, reducing the solvent content by dehydration has enabled atomic resolution structures to be obtained from previously poorly diffracting crystals (Heras *et al.*, 2003 ▶; Yap *et al.*, 2007 ▶; Sam *et al.*, 2006 ▶; Haebel *et al.*, 2001 ▶; Koch *et al.*, 2004 ▶; Rojviriya *et al.*, 2011 ▶; Pauwels *et al.*, 2005 ▶; Stocker *et al.*, 2005 ▶). Changing the crystal solvent content may alter the unit-cell parameters and in some cases even lead to major lattice rearrangements including space-group changes (Jenni & Ban, 2009 ▶; Bailly *et al.*, 2009 ▶). This modification of crystal packing relies on the flexibility of the crystallized macromolecule within the crystal. The induced changes range from minor local rearrangements to more dramatic domain rearrangements exploiting alternative conformations of the macromolecule (Esnouf *et al.*, 1998 ▶; Bowler *et al.*, 2007 ▶; Sanchez-Weatherby *et al.*, 2009 ▶; Einstein *et al.*, 1963 ▶; Huxley & Kendrew, 1953 ▶).

Crystals can be dehydrated in a number of ways. The simplest approach is to expose the crystal directly to air (Bragg & Perutz, 1952 ▶). More gentle approaches involve soaking the crystal in a dehydrating solution or equilibrating the crystallization drop against a dehydrating solution prior to crystal harvesting (Charron *et al.*, 2002 ▶; Mi *et al.*, 2004 ▶; Timasheff & Arakawa, 1988 ▶; Abergel, 2004 ▶; Douangamath *et al.*, 2013 ▶). The dehydration environment can be carefully controlled using dehydration chambers (Huxley & Kendrew, 1953 ▶; Pickford *et al.*, 1993 ▶) or by the Micro-RT system (Skrzypczak-Jankun *et al.*, 1996 ▶; Kalinin *et al.*, 2005 ▶) in which crystals can be kept at the desired relative humidity (RH) during room-temperature data collection.

A more rational and controlled method for studying the impact of dehydration is offered by automated humidity controllers, which provide a stream of humid air to envelop a crystal mounted on a standard sample holder (Kiefersauer *et al.*, 2000 ▶; Sjögren *et al.*, 2002 ▶). Coupled with an X-ray source, they allow the evaluation of serial variations in RH and the identification of any transition(s) that could be exploited to obtain improved crystal packing. One available system is the HC1 humidity controller (ARINAX, France), which represents the most recent development in the field (Sanchez-Weatherby *et al.*, 2009 ▶). It provides a simple and versatile way of maintaining crystals at the desired RH and is available at several synchrotrons and laboratories worldwide.

For a humidity-controlled dehydration experiment, it is essential for the crystal to be exposed directly to the humid air stream to control the rate of hydration change and hence improve reproducibility. This can be achieved by mounting the crystal on a Kapton mesh and wicking away residual mother liquor by gently tapping the sample with a tissue. This technique is risky and crystals are susceptible to physical damage during the process.

A recently developed tool for crystal harvesting, commercially known as Crystal Catcher (Kyodo International Inc.), makes use of adhesives mounted on a quasi-SPINE standard pin (Kitatani, Sugiyama *et al.*, 2008 ▶). There is a choice of two adhesives: ‘Type A’ (known as ‘Aqua’ by the manufacturer) for adhering crystals grown in aqueous solutions (used in the CT-100 and CT-300 mounts) and ‘Type B’ (known as ‘Salt’ by the manufacturer) for crystals grown in organic solvents (used in the CT-200 and CT-400 mounts). To harvest crystals with the Crystal Catcher tool, a small amount of adhesive is extruded from a 20 mm long hollow pin mounted on a magnetic base that is compatible with standard goniometers. Crystals stick to the adhesive upon contact, allowing them to be readily removed from the mother liquor. This loop-free approach is very attractive as it allows transfer of crystals from the drop with minimal excess liquid (Kitatani, Adachi *et al.*, 2008 ▶; Kitatani *et al.*, 2009 ▶). For cryocrystallography this crystal-mounting approach can eliminate the requirement for cryoprotectants (Pellegrini *et al.*, 2011 ▶). Additionally, reduced X-ray absorption and background scattering from the sample can benefit data collection for anomalous phasing (Lewis & Rees, 1983 ▶). The Crystal Catcher may also be a useful tool for mounting crystals for dehydration experiments as it would eliminate the need for crystal wicking.

Here, we present a thorough description of the use of the Crystal Catcher in conjunction with the HC1 installed on beamline I02 at the Diamond Light Source.

## 2. Materials and methods   

### 2.1. Crystallization of samples   

Holoferritin from horse spleen (Sigma–Aldrich catalogue No. F4503) was crystallized by mixing 3 µl 40 mg ml^−1^ protein solution (diluted with distilled H_2_O) with 2 µl reservoir solution consisting of 0.1 *M* NaCl, 0.8 *M* ammonium sulfate, 10 m*M* CdSO_4_, 25%(*v*/*v*) glycerol. Bipyramidal crystals (∼200 × 200 × 200 µm) appeared in 2 d from sitting-drop plates at 293 K.

Proteinase K from *Tritirachium album* (Sigma–Aldrich catalogue No. P2308) was crystallized by mixing 1 µl 20 mg ml^−1^ protein solution in 25 m*M* Na HEPES pH 7.0, 100 µ*M* PMSF with 1 µl reservoir solution consisting of 0.1 *M* Bis-Tris pH 5.5, 0.65 *M* LiCl. Bipyramidal crystals (∼100 × 100 × 200 µm) appeared overnight from sitting-drop plates at 293 K.

Thaumatin from *Thaumatococcus daniellii* (Sigma–Aldrich catalogue No. T7638) was crystallized by mixing 2 µl 40 mg ml^−1^ protein solution with 1 µl reservoir solution consisting of 50 m*M* ADA pH 6.8, 0.6 *M* potassium/sodium tartrate, 1.5 m*M* 2,2′-dinitro-5,5′-dithiobenzoic acid (DTNB), 20%(*v*/*v*) glycerol. Bipyramidal crystals (∼100 × 100 × 200 µm) appeared in one week from sitting-drop plates at 293 K.

Thermolysin from *Bacillus thermoproteolyticus* (Calbiochem catalogue No. 58656) was crystallized by mixing 1 µl 100 mg ml^−1^ protein solution in 50 m*M* Na MES pH 6.0, 45%(*v*/*v*) DMSO with 1 µl reservoir solution consisting of 1.96 *M* ammonium sulfate. Hexagonal, rod-like crystals (∼200 × 200 × 400 µm) appeared in one week from sitting-drop plates at 293 K.

Trypsin from bovine pancreas (Sigma–Aldrich catalogue No. T1426) was crystallized by mixing 1 µl 60 mg ml^−1^ protein solution in 10 m*M* CaCl_2_, 83 m*M* benzamidine with 1 µl reservoir solution consisting of 0.1 *M* Tris–HCl pH 8.5, 2 *M* ammonium sulfate. Cuboid crystals (∼50 × 50 × 50 µm) appeared overnight from sitting-drop plates at 293 K.

Hen egg-white lysozyme (Hampton Research catalogue No, HR7-110) was crystallized by mixing 1 µl 40 mg ml^−1^ protein solution with 1 µl reservoir solution consisting of 50 m*M* sodium acetate pH 4.6, 30%(*w*/*v*) PEG MME 5000, 1 *M* NaCl. Cubic crystals (∼100 × 100 × 400 µm) appeared in a less than 1 h from sitting-drop plates at 293 K.

Glucose isomerase from *Streptomyces rubiginosus* (Hampton Research catalogue No. HR7-102) was dialysed into 25 m*M* Tris–HCl pH 7.5 and was then crystallized by mixing 2 µl of 25 mg ml^−1^ protein with 2 µl reservoir solution consisting of 10%(*w*/*v*) PEG 400, 20%(*w*/*v*) glucose, 50 m*M* MgCl_2_, 0.1 *M* HEPES pH 7.0. Rhombic dodecahedral crystals (∼100 × 100 × 30 µm) appeared within 2 d.

P4 DNA crystals (a gift from James Hall) were obtained as described in Hall *et al.* (2011 ▶), resulting in cubic crystals (∼50 × 50 × 50 µm).

Protein kinase PIM1 crystals were obtained as described in Kumar *et al.* (2005 ▶), resulting in cubic rods (∼25 × 25 × 200 µm).

### 2.2. Crystal harvesting   

Crystals were harvested using the Crystal Catcher tool (Kyodo International; Supplementary Video S1^1^) or Kapton micro-fabricated meshes (MiTeGen, M3-L18SP-A1)

The Crystal Catcher tool can use either metallic pins (CT-100 or CT-200) or glass capillaries (CT-300 or CT-400). The amount of adhesive extruded by the tool is finely controlled by slowly rotating its metallic base counter-clockwise. Usually, 5° or less is sufficient to extrude 50–100 µm^3^ of adhesive. After the experiments the crystals were removed either with the cleaning tool (CL-100) or, more efficiently, by sliding a paper tissue from the base of the pin to the crystal (Supplementary Video S2).

Swelling and preconditioning experiments on adhesive ‘Type A’ were performed by mounting the metallic base on a static magnetic holder and exposing it to an RH of 99.9% *via* an offline setup of the HC1. Images were collected using a digital microscope coupled with the HC1 control software that provides real-time measurement of the drop size. Estimation of the equilibrium RH for the two types of adhesive was obtained by varying the RH of the HC1 until the drop size remained stable over time.

### 2.3. Dehydration experiments and data collection   

Diffraction experiments were carried out by mounting samples directly on the goniometer of beamline I02 at the Diamond Light Source and maintaining them in a controlled-humidity environment with the HC1 at room temperature (∼293 K). At each RH, four diffraction images (0.1 s per frame, 1° per frame, 1% transmission for a 12.658 keV beam at 3.2 × 10^12^ photons s^−1^) were recorded on a Pilatus 6MF detector (Dectris) and indexed using *MOSFLM* (Leslie, 2006 ▶).

Full diffraction data sets were collected from crystals of glucose isomerase and thermolysin. The first data set from each crystal was taken at room temperature using data-collection parameters chosen to maximize data completeness and to minimize radiation damage. Samples were then cryocooled within 2 s by swapping the HC1 nozzle with the cryojet using the automated procedure available at the beamline (Supplementary Video S3). Following this, a second data set was obtained at 100 K using a different position on the crystal and the same data-collection parameters as for the room-temperature data set.

X-ray diffraction images were indexed, scaled and integrated with *xia*2 (Winter *et al.*, 2013 ▶). The analysis of the background scattering contribution for the different types of sample holder was performed using the *Data Analysis WorkbeNch* package (*DAWN*; http://www.dawnsci.org).

## 3. Results   

### 3.1. Sample harvesting with the Crystal Catcher   

Crystal Catcher is the commercial name for a tool designed for crystal harvesting. It consists of a metallic, hollow pin (200 µm outer diameter/100 µm inner diameter) from which an adhesive is extruded to anchor a protein crystal. A magnetic base is used to secure the tool either to a magnetic wand, during harvesting procedures, or to a goniometer head, during X-ray diffraction experiments.

Two types of adhesives are currently available that were designed to mimic the precipitant conditions of the crystals to be harvested. The Crystal Catcher CT-100 is a pin containing an adhesive for harvesting crystals from aqueous solutions, and CT-200 contains an adhesive designed to work for crystals grown in organic solvents and viscous solutions (Kitatani, Adachi *et al.*, 2008 ▶). A modified sample holder containing the same adhesives (CT-300 and CT-400, respectively) uses a glass capillary instead of the metallic pin, reducing the inner diameter of the extruded glue to approximately 50 µm. This uses less adhesive and makes it easier to harvest smaller crystals (<50 µm; Kitatani *et al.*, 2009 ▶).

An initial qualitative approach was performed to assess the ease of handling and manual mounting of different macromolecular crystals. In general, at least one of the two adhesives proved applicable to harvesting the samples in the test set. However, despite being grown in aqueous and non-viscous precipitants, most samples did not stick to mounts containing the ‘Type A’ adhesive. On the other hand, the ‘Type B’ adhesive found in the CT-200 pin proved much more effective: with one exception, all of the test crystals stuck to the droplet of adhesive and were safely mounted on the goniometer for data collection. Only glucose isomerase crystals did not stick to the ‘Type B’ adhesive, but these were easily mounted with the ‘Type A’ adhesive (Table 1 ▶).

**Figure 1:**
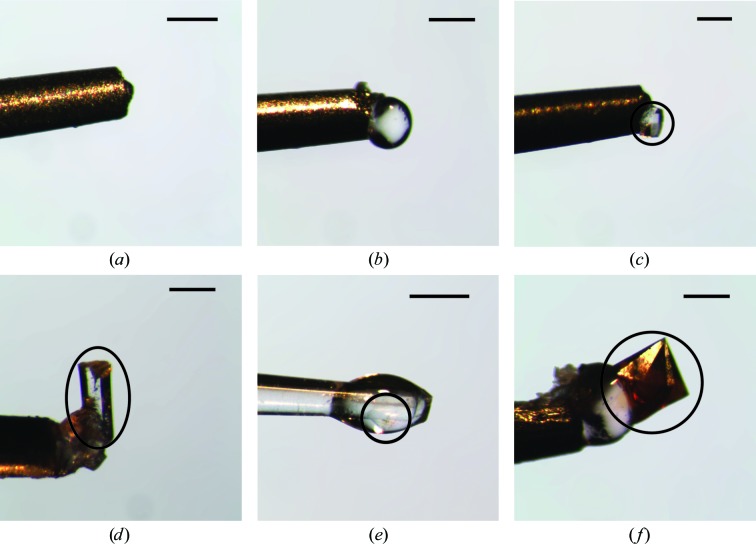
Examples of crystal harvesting. Metal pins loaded with a small (*a*) and large (*b*) amount of the ‘Type A’ adhesive and subsequently used to harvest a small lysozyme crystal (*c*) and a large thermolysin crystal (*d*). Example of a trypsin crystals mounted with a CT-400 glass pin (*e*) and holoferritin mounted after pre-conditioning the adhesive to the correct humidity (*f*). The scale bars represent 200 µm and a black circle indicates the location of the crystals.

The glass capillary version of the Crystal Catcher proved to be particularly useful when harvesting crystals smaller than 100 µm and was essential if the samples were smaller than 50 µm. The manufacturer recommends using the minimum amount of adhesive possible (Fig. 1 ▶), as an excess makes it difficult to locate samples once they are mounted at the sample position. This is true for very small transparent samples, but large or coloured crystals are easily located even when embedded in a large drop of adhesive.

Despite the fact that the ‘Type B’ adhesive was appropriate for mounting most of the test crystals, the ease of harvesting crystals was very sample-dependent. As with other mounting techniques, the main challenge is harvesting delicate crystals, especially if they adhere to the crystallization plate. This can be overcome when working by hand but would be difficult to automate. This is in agreement with a recent report, which states that this tool is unsuitable for the automation of crystal harvesting using robotics (Viola *et al.*, 2011 ▶).

### 3.2. Crystal Catcher and relative humidity variations   

To minimize osmotic stress on the crystals during dehydration experiments, the initial RH of the air stream must be equilibrated with that of the mother liquor. This is achieved by monitoring the size variation of a drop of reservoir solution and adjusting the RH provided by the HC1 to maintain a constant drop size (Russi *et al.*, 2011 ▶; Sanchez-Weatherby *et al.*, 2009 ▶). When crystals are harvested using an adhesive, consideration should be given to the chemical composition of this adhesive as a factor influencing the system. For this reason, the initial equilibrium RH of both adhesives was determined. In addition, the adhesive behaviour when exposed to increased or reduced RH was monitored.

A large amount of adhesive was extruded (*i.e.* more than that required for harvesting crystals) to generate a round drop standing out from the metallic pin, and this pin was then mounted on the goniometer (Fig. 1 ▶ *b*). The initial RH for both adhesives was determined by measuring the diameter of the extruded adhesive, as is usually performed with crystallization reservoir solutions (Russi *et al.*, 2011 ▶; Sanchez-Weatherby *et al.*, 2009 ▶). The ‘Type B’ adhesive, designed for organic solvent and viscous solutions, was determined to have an initial equilibrium RH of 99.9 ± 0.1%. This is in accordance with the RH values calculated using Raoult’s law for high-molecular-weight PEG-based mother liquors (Wheeler *et al.*, 2012 ▶).

The ‘Type A’ adhesive was determined to have an initial equilibrium RH of 83.0 ± 0.5%. This measurement also matched expectations, since the RH of aqueous salt solutions is observed to be between 80 and 95% and is typically lower than for organic precipitants. The range of RH values at which crystals grow means that the equilibrium point of the Crystal Catcher adhesives will rarely match the initial values required by a dehydration experiment. For example, lysozyme and glucose isomerase (grown at high RH, *i.e.* between 99.9 and 97%) are best harvested with the ‘Type A’ adhesive (stable at 83%). If samples are mounted under such conditions, the glue will require a transition time to equilibrate prior to the start of any experiment. During this process, or when a dehydration experiment is carried out, the humidity changes will affect both the adhesive and the sample, and may result in uncontrolled movement, crystal fractures or even chemical modifications.

**Figure 2:**
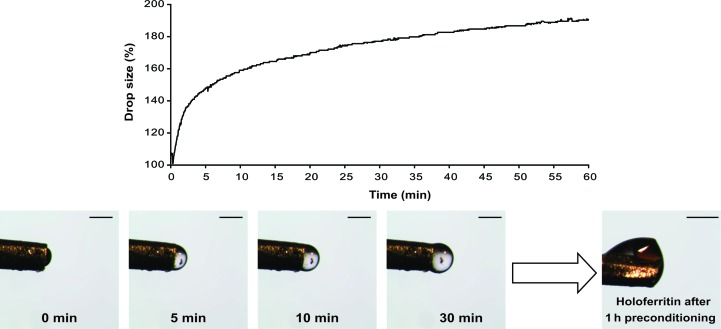
Adhesive expansion during preconditioning. Plot of the increase in drop size over time of the ‘Type A’ adhesive upon hydration at 99.8% RH. Calculations were performed *via* the HC1 control software (Sanchez-Weatherby *et al.*, 2009 ▶) and adhesive images are shown from selected time points. The lower right picture displays a holoferritin crystal successfully harvested after preconditioning the ‘Type A’ adhesive for 60 min. The scale bars represent 200 µm.

To assess these effects, a droplet of ‘Type A’ adhesive (stable at 83% RH) was placed directly into an airstream with 99.8% RH and its size was monitored over time (Fig. 2 ▶). This demonstrated the changes that might occur after mounting a sample and enabled measurement of the rate of expansion of the adhesive. As shown in Fig. 2 ▶, the drop of adhesive swells dramatically within the first 5 min, increasing its radius by more than 50%. After a first exponential phase, the curve profile becomes less steep and approaches a plateau after 30 min.

It was also observed that preconditioning the adhesive to the appropriate RH of the samples to be harvested helped in the harvesting of samples that did not naturally stick to the adhesive. For example, holoferritin crystals did not stick to native ‘Type A’ adhesive, but when this was preconditioned at 99.8% RH for 30 min crystals could be lifted easily (Fig. 2 ▶). Nonetheless, preconditioning softens and swells the adhesive and it is not yet clear whether the expected shrinkage of a hydrated adhesive would affect the integrity of the crystal during a dehydration experiment.

The increase in drop size of the ‘Type A’ adhesive upon exposure to high RH is probably caused by a combined effect of the adhesive swelling and additional adhesive withdrawal from the capillary, as the drop expansion is not completely reversible, *i.e.* reduction of the RH around a swollen adhesive does not reduce it to the original size (data not shown). This could only be confirmed by measuring the same effect on a drop of adhesive alone (*i.e.* not mounted on the Crystal Catcher pin), but this has not been tested. The ‘Type B’ adhesive (which has a 99.9% RH) was also tested by lowering the RH to 75%, but neither contractions of the drop nor movements of mounted crystals were detected (data not shown).

### 3.3. Benefits of glass capillary-mounted adhesives   

**Figure 3:**
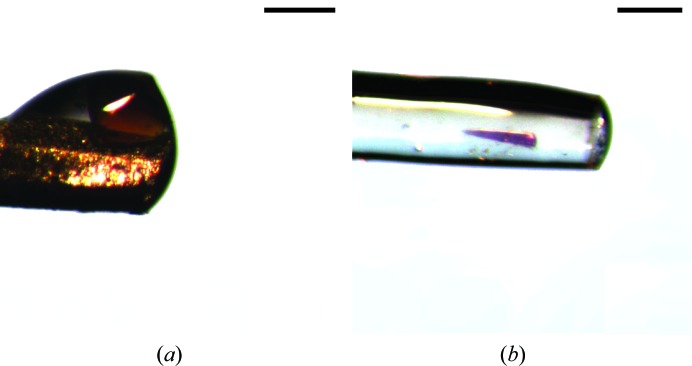
Protein crystals adhered to both metallic and glass pins. (*a*) A holoferritin crystal on the external side of a CT-100 metallic mount and (*b*) a coloured PIM1 crystal stuck to the side of a CT-300 glass mount (*b*). The scale bars represent 200 µm.

As discussed earlier, the position of crystals can change as the mechanical properties of the adhesive are modified during humidity-control experiments. This was particularly evident in the swollen ‘Type A’ adhesive described above. Despite this, during a dehydration experiment the adhesive adapts rapidly to a new RH (within approximately 1 min). Given the high viscosity of these adhesives, crystals do not move after this time, so it is not normally a problem during dehydration experiments. However, as the direction and extent of crystal movement cannot be controlled this may complicate attempts to collect full data as it may limit the accessible rotation angle owing to the metal pin blocking the X-ray beam (Fig. 3 ▶ *a*).

In such cases, it is very convenient to use the glass capillary mounts (CT-300 or CT-400). They are transparent, making it possible to locate the crystal, and glass has a relatively low X-ray absorption coefficient, which allows data to be collected even when the crystal is located on the wall of the capillary (Fig. 3 ▶ *b*).

Glass capillary-based Crystal Catcher pins are best suited for harvesting small crystals owing to their smaller diameter and transparency. However, these pins are more fragile and crystals frequently adhere to the side of the capillary. Their use requires more careful handling and greater care in alignment when collecting data to avoid additional background and/or absorption effects.

### 3.4. Background diffraction from different crystal holders   

**Figure 4:**
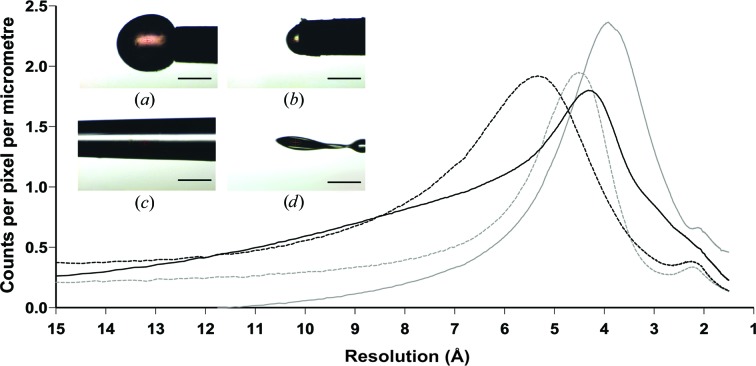
The background scattering from the adhesive compared with parafin. Graph showing the radial profile of the scattering density (averaged radial counts per pixel per micrometre of material in the X-ray beampath) of different mounts. The insets show the object producing the scattering: a large ‘Type A’ adhesive droplet (*a*, solid black line), a small ‘Type B’ adhesive droplet (*b*, dashed black line), ‘Type A’ adhesive inside a CT-300 glass capillary (*c*, solid grey line) and a nylon loop with paraffin (*d*, dashed grey line). The scale bars represent 200 µm and the beam was collimated to 50 × 25 µm.

The X-ray beam interacts with all sample holders and the retained mother liquor, and this has an effect on the diffraction images. Background scatter was compared between the Crystal Catcher and other commonly used sample holders. Five X-ray diffraction images for each experiment were averaged and the air background was subtracted using *DAWN* (http://www.dawnsci.org). Radial plots of intensity (Fig. 4 ▶) show relatively low background scattering for the ‘Type A’ and ‘Type B’ adhesives (solid and dashed black lines, respectively), accounting for a maximum of 1.8–1.9 counts per micrometre pathlength of adhesive. A similar profile is obtained for a nylon loop filled with paraffin oil (dashed grey line). This means that for the sample holders tested here, the background intensity is proportional to the thickness of the support illuminated by X-rays.

For humidity-controlled experiments, crystals are usually centred such that the beam does not hit the adhesive, so the contribution from the adhesive is negligible. Even when the adhesive cannot be avoided, a convenient amount can normally be extruded so that the background scattering is kept to a minimum (approximately 50 µm in thickness). This is not the case when collecting through the glass capillary (Fig. 4 ▶, solid grey line) as, given its minimum thickness of 200 µm, it will always have a higher background. This is still acceptable when performing a humidity-controlled experiment aimed at defining the change in unit-cell parameters upon dehydration.

The main advantage of the Crystal Catcher is that crystals can be mounted without any surrounding mother liquor and with minimal support, thereby reducing background scattering to a minimum. In addition to assessing background, X-ray fluorescence scans were performed on both adhesives to evaluate the presence of trace elements. Neither of the adhesives emitted fluorescence in the 2–16 keV range (data not shown). This eliminates the presence of any major contaminant of atomic number greater than 15, confirming the suitability of both adhesives for use in phasing experiments involving typically used anomalous scattering elements.

### 3.5. Data collection with the Crystal Catcher   

The Crystal Catcher is marketed as a useful tool for harvesting samples for X-ray diffraction experiments and can be used at cryogenic temperatures. Tests of both adhesives with several crystal types (Table 2 ▶) at 293 K using the HC1 mounted on beamline I02 at the Diamond Light Source confirm comparable data-collection statistics to those obtained for similar crystals mounted on loops or meshes. Unit-cell parameters are as expected for room-temperature samples, although mosaic spread values are not as low as in previous reports (Kitatani, Adachi *et al.*, 2008 ▶), possibly owing to intrinsic sample variation.

Cryocooling of adhesive-mounted samples was achieved by automatically swapping the HC1 nozzle with that of the Cryojet (Oxford Instruments). This procedure takes 2 s at beamline I02 and visual inspection using the beamline on-axis microscope confirmed that the temperature change did not affect the adhesive. The quality of the diffraction pattern of cryocooled crystals is often different to that at room temperature and the effect of cooling is mainly sample-dependent. Glucose isomerase crystals diffracted better when cryocooled, whereas thermolysin crystals diffracted worse (and were often damaged) when cooled in this way. Control experiments using standard meshes and removing mother liquor by wicking show identical patterns of behaviour and similar data quality to those obtained with the Crystal Catcher.

If the bare adhesive is hydrated (*e.g.* ‘Type A’ kept at 99% RH) prior to cryocooling, ice rings appear in the X-ray diffraction images. This is probably owing to the inability of the hydrated resin to prevent the formation of crystalline ice by the water it has absorbed. When preparing samples for cryogenic data collection it is advisable to keep the part of the crystal that is to be illuminated with the X-ray beam outside the adhesive.

Automatic cryogenic sample mounting with these pins is not currently possible at the Diamond Light Source, as the design of the Crystal Catcher does not comply with the required SPINE standard (Cipriani *et al.*, 2006 ▶). Since the pin cap fits SPINE vials, it may be possible to mount these samples at other facilities with different sample changers.

### 3.6. HC1 experiments with the Crystal Catcher   

**Figure 5:**
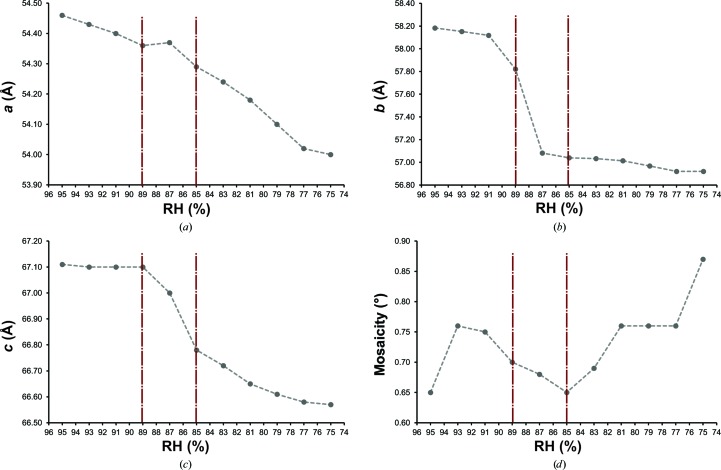
Dehydration experiment on an orthorhombic trypsin crystal. Plots showing changes of the *a*, *b* and *c* unit-cell parameters (*a*, *b*, *c*) as well as mosaic spread (*d*) as a function of decreasing relative humidity for an orthorhombic trypsin crystal. Vertical lines indicate transition points for which the trend of one or more of the monitored parameters change as the RH changes.

To assess the usefulness of adhesive crystal mounting for controlled dehydration experiments at the beamline, we monitored the RH dependence of unit-cell parameters and mosaicity for three types of crystals harvested with the CT-200 pin and one with the CT-100 pin (Fig. 5 ▶ and Supplementary Figs. S1–S3).

Tetragonal lysozyme samples (Supplementary Fig. S1) were used as the first test sample. Upon decreasing the RH stepwise from 99 to 70%, the *a* and *b* unit-cell parameters of the crystals gradually reduced in length. At an RH of 80%, a transition point was identified at which the *c* unit-cell parameter also started decreasing and the mosaic spread values reach a minimum. This is probably a reflection of increased lattice order as excess solvent is removed. This more compact packing, which is then perturbed by further dehydration, is consistent with previous observation of crystals dehydrated in plates (Pierre Aller, personal communication). This type of transition within a range of RH values usually allows the identification of points at which an increase in resolution is to be expected. Once such a transition point has been detected, fresh crystals should be dehydrated to this RH point. Data collection can then be tested with cryocooled dehydrated crystals, where radiation damage will be less significant.

Other crystals tested included two crystal forms of trypsin: an orthorhombic and a trigonal form (present in the same crystallization drops). Both undergo a structural transition, albeit at different values of RH. In the case of the ortho­rhombic trypsin crystals (Fig. 5 ▶), the *b* and *c* unit-cell parameters remain stable above a RH of 88%, decreasing with dehydration beyond this point. Analysis of the mosaicity values suggest that, added to the decrease in all unit-cell parameters with dehydration, there is an optimal ordered state at around a RH of 85% that also coincides with a change in the rate of change for the *c* unit-cell parameter.

For the trigonal trypsin crystals (Supplementary Fig. S2), the transition occurs at 91% RH, where the mosaicity is at a minimum. When reducing the RH further, the decrease in the *a* and *b* unit-cell parameters is accompanied by a lengthening of the *c* axis. In this case, it is suggested that data collection from cryocooled crystals be explored with crystals dehydrated to 91 and 87% RH to assess each transition identified

The final test samples were P4 DNA crystals (Supplementary Fig. S3), which upon decreasing the RH from 86 to 65% showed a progressive decrease of all unit-cell parameters with a dip in the mosaicity value that could indicate a more stable point around 74%.

### 3.7. Dehydration buffering effect of the Crystal Catcher   

P4 DNA crystals have been studied using Kapton meshes (J. Hall *et al.*, manuscript in preparation) and the changes observed then, although similar to those presented here, were not identical. One explanation for these differences is that in the present study the crystals are partially engulfed by adhesive, potentially affecting the dehydration process.

**Figure 6:**
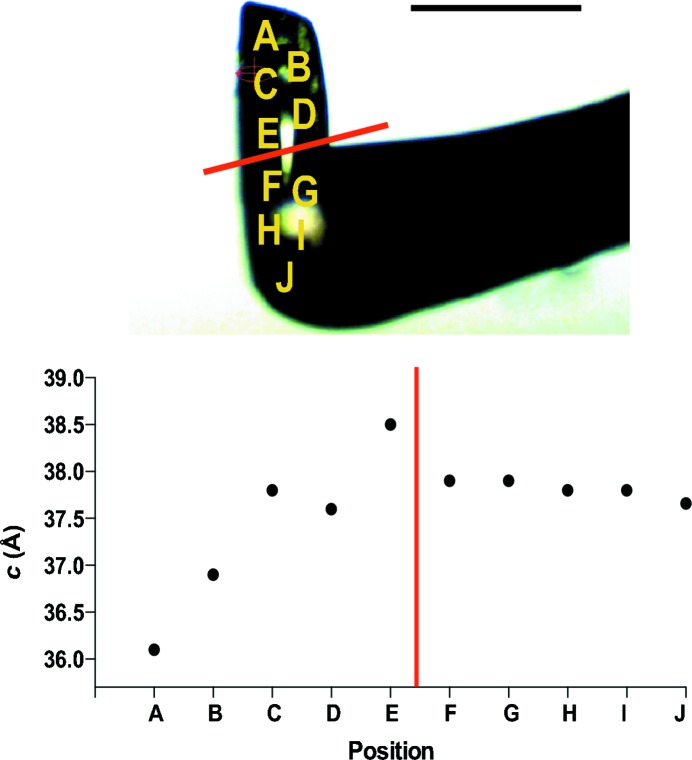
Crystal Catcher adhesive protects crystals from dehydration. Diagram showing the positions where data were collected on a lysozyme crystal undergoing dehydration partially engulfed in adhesive (top). Graph of the resulting *c* unit-cell parameter as a function of the position after 5 min of equilibration at 70% RH (bottom). Crystal positions directly exposed to the humid air stream, *i.e.* outside the adhesive (positions A and B), are more responsive to RH reduction and have shorter *c* unit-cell axis than those embedded within the adhesive (positions C–J). The scale bar represents 200 µm.

To investigate the effect of adhesive on the dehydration process, data were collected from a rod-shaped lysozyme crystal harvested with a CT-200 pin using an amount of adhesive such that half of the crystal was embedded in it (Fig. 6 ▶). A focused beam, collimated to 50 × 25 µm using beam-defining slits, was used to collect data at different positions on the crystal within 5 min of an instant dehydration step (mounting directly at 70% RH).

For regions of the crystal outside the adhesive, the *a* and *b* unit-cell parameters were 77 Å and the *c* unit-cell parameter was 37 Å, matching the expected values for lysozyme at 70% RH. For regions inside the adhesive, all three unit-cell parameters were larger and were more similar to those observed previously at the initial humidity of the experiment, *i.e.* 99% RH (Supplementary Fig. S1).

Changes were also monitored over a longer time at the regions of the crystal surrounded by adhesive, and they demonstrated that the area embedded in adhesive equilibrates to the expected unit-cell parameter values within 15–20 min (data not shown). This experiment confirms that any excess adhesive surrounding the crystal may mask dehydration effects, distorting the interpretation of changes during a dehydration experiment. This result is consistent with observations by other groups (Baba *et al.*, 2013 ▶), in which glue-coated crystals were also protected from RH changes, and slow cell rearrangements occurred within half an hour from the beginning of the dehydration process. This time lag complicates the interpretation of successive dehydration events. Mounting the crystal with the minimum of adhesive and collecting data from the part most exposed to air reduces this protective effect and allows a better estimation of unit-cell parameter changes according to the hydration state. Alternatively, surrounding samples with a thin layer of adhesive could prove beneficial in very delicate systems where the standard approach may not be gentle enough to trap certain dehydration intermediates.

## 4. Conclusions   

The collection of X-ray diffraction patterns from macromolecular crystals requires the sample to be mounted on a goniometer that rotates the sample during data collection. This is traditionally achieved through sample holders such as nylon loops that are used to harvest crystals from their mother liquor (Garman, 1999 ▶). Besides these, Kapton meshes and loops with low background scattering are also available on the market (Thorne *et al.*, 2003 ▶). In all of the above cases, a thin layer of mother liquor surrounds the samples, keeping the crystals in a near-native environment but also resulting in additional background scatter. As an alternative, samples can be maintained in their own native environment by mounting the entire crystallization plate and collecting diffraction images *in situ* (Axford *et al.*, 2012 ▶), but this also causes higher background scattering owing to the plastic support. The Crystal Catcher tool described herein offers a valuable alternative to all these techniques, since the crystals can be mounted directly without excess surrounding liquid. Naked crystals can be harvested and mounted even by relatively inexperienced crystallographers. Despite initial results that were not very promising (Viola *et al.*, 2011 ▶), automated harvesting of crystals with no surrounding mother liquor has been proposed for robotic devices (FMP Products Inc., Greenwich, Connecticut, USA) using the Crystal Catcher tool and may be extended to other systems such as CrystalDirect (Cipriani *et al.*, 2012 ▶).

The currently available Crystal Catcher adhesive formulations and pin formats provide means to harvest crystals of different types and sizes. However, the task is, depending on the samples, not always straightforward and requires practice. The stability of both the ‘Type A’ and ‘Type B’ adhesives at different RH levels reflects their different chemical composition, which mimics that of the crystallization solution. In general, it was determined that the ‘Type B’ adhesive, which was designed for PEG-based precipitants, often work well with salt-based precipitants. Considering this in terms of chemical potential, osmolarity matching may be the driving force for the selection of the right adhesive. This is confirmed by our experiments on holoferritin crystals, which could not be attracted by the ‘Type A’ adhesive unless the adhesive was preconditioned to the RH of the ‘Type B’ adhesive, thus modifying the water concentration within the adhesive. The lack of intermediate adhesives covering the range between 99.9% RH of the ‘Type B’ adhesive and 83% RH of the ‘Type A’ adhesive makes it difficult to rationalize this observation. Furthermore, as the formulation of these adhesives is proprietary, this does not allow more detailed testing and optimization. An alternative to reformulating the adhesives could be to precondition the adhesive (by keeping it at a controlled RH) prior to every harvest, but this would be a slow process. This highlights the need for a more refined choice of adhesive or a mixing kit to allow users to optimize the composition, as has been suggested for other glue-based supports (Baba *et al.*, 2013 ▶).

The most important difference between the Crystal Catcher and standard sample holders, such as loops and meshes, is that adhesion is achieved by the Crystal Catcher adhesive touching the sample directly without a layer of mother liquor to shield this contact. Since the adhesive is not chemically inert, it could induce chemical modification of the crystal in direct contact with it. The different composition and water content of the adhesives may also lead to direct hydration/dehydration of the crystal. This is also reflected in the protective effect observed when RH variations are buffered by the adhesive in the embedded regions of the crystal. To minimize these chemical effects, it is best practice to centre the X-ray beam position on the part of the crystal furthest from its point of contact with the adhesive.

The swelling observed when the ‘Type A’ adhesive was incubated at 99.8% RH highlights the susceptibility of this adhesive to hydration, which often leads to a movement of the crystal during the first 5 min of equilibration at a specific RH. However, crystal movement does not hamper the suitability of the Crystal Catcher for dehydration experiments, since these experiments usually require 5–15 min incubation time at each RH. If crystal movement hinders data collection, glass pins may be used to ensure that crystals can be exposed to X-rays. This demonstrates that the Crystal Catcher or similar adhesive sample holders are valuable for HC1 experiments and are an ideal method to allow fast mounting and reliable detection.

If the correct amount of adhesive is used (*i.e.* as little as possible to harvest the crystal), no background scatter from the adhesive is observed in the diffraction pattern obtained. If some adhesive is required for harvesting, the background given by the Crystal Catcher is comparable to that of an equivalent amount of mother liquor or cryoprotectant. These properties make the Crystal Catcher ideal for both room and cryogenic temperature data collection. Furthermore, by totally depleting the mounted crystals of mother liquor, the Crystal Catcher may provide a good alternative to standard cryoprotectants. This is supported by the observations of crystals cryocooled by switching from the HC1 to a cryostream, but will require further testing.

Incompatibility with the SPINE standard pins may limit the uptake of the Crystal Catcher across Europe for cryocrystallography and further standardization will be required to allow large-scale uptake.

In conclusion, the Crystal Catcher is a versatile tool for crystal harvesting and post-crystallization optimization. In combination with the HC1 it provides the lowest possible background and allows high-quality data collections, which are crucial for challenging crystallographic projects.

## Supplementary Material

Supplementary Video S1. Crystal harvesting using the Crystal Catcher.. DOI: 10.1107/S1399004714014370/gm5033sup1.wmv

Supplementary Video S2. Removal of the crystals from Crystal Catcher using the cleaning tool (CL-100). DOI: 10.1107/S1399004714014370/gm5033sup2.wmv

Supplementary Video S3. Cryocooling of crystals by automated swapping HC1 nozzle to cryojet.. DOI: 10.1107/S1399004714014370/gm5033sup3.wmv

Supporting Information.. DOI: 10.1107/S1399004714014370/gm5033sup1.pdf

## Figures and Tables

**Table 1:** Use of the ‘Type A’ and ‘Type B’ adhesives to harvest protein or DNA crystals grown in the presence of different precipitant solutions

Crystal	‘Type A’ adhesive	‘Type B’ adhesive	Main precipitant
Lysozyme	Yes	Yes	1 *M* NaCl, 20% PEG MME 5000
Holoferritin	Yes (if preconditioned)	Yes	0.8 *M* ammonium sulfate
Proteinase K	No	Yes	0.65 *M* LiCl
Trypsin	No	Yes	2 *M* ammonium sulfate
Thermolysin	Difficult	Yes	1.96 *M* ammonium sulfate
Thaumatin	No	Difficult	0.6 *M* potassium/sodium tartrate
Glucose isomerase	Yes	No	20% PEG 400
P4 DNA	Yes	n.a.	35% MPD
PIM1 kinase	No	Yes	0.8 *M* sodium acetate

**Table 2:** Comparison between data sets from crystals harvested with the Crystal Catcher and Kapton meshes Data from glucose isomerase and thermolysin crystals mounted with the two supports were collected both at room temperature (293 K) and at cryo-temperature (100 K). Values in parentheses are for the highest resolution shell.

	Glucose isomerase				Thermolysin			
Relative humidity (%)	94.00				97.00			
Holder/adhesive	CT-100/‘Type A’		Mesh		CT-200/‘Type B’		Mesh	
Temperature (K)	293	100	293	100	293	100	293	100
Space group	*I*222	*I*222	*I*222	*I*222	*P*6_1_22	*P*6_1_22	*P*6_1_22	*P*6_1_22
*a* (Å)	94.14	92.99	94.16	92.99	93.67	93.04	93.70	93.15
*b* (Å)	99.37	98.68	99.38	98.33	93.67	93.04	93.70	93.15
*c* (Å)	102.94	102.69	102.96	102.64	130.36	127.56	130.63	128.31
α = β (°)	90	90	90	90	90	90	90	90
γ (°)	90	90	90	90	120	120	120	120
Mosaicity (°)	0.142	0.139	0.075	0.084	0.035	0.176	0.017	0.182
Resolution (Å)	40.4–2.0 (2.1–2.0)	40.1–2.0 (2.1–2.0)	39.3–2.0 (2.1–2.0)	40.0–2.0 (2.1–2.0)	46.8–1.8 (1.9–1.8)	46.5–3.2 (3.3–3.2)	46.9–1.8 (1.9–1.8)	64.2–3.2 (3.3–3.2)
Completeness (%)	99.1 (99.6)	99.1 (98.8)	99.7 (99.1)	99.5 (98.3)	97.7 (98.5)	95.5 (96.1)	100.0 (99.9)	100.0 (100.0)
Multiplicity	3.7 (3.8)	3.6 (3.8)	3.7 (3.8)	3.7 (3.8)	11.0 (11.1)	10.7 (10.8)	10.7 (10.9)	10.1 (10.4)
〈*I*/σ(*I*)〉	25.9 (15.9)	29.5 (20.8)	39.1 (22.0)	30.8 (18.0)	18.0 (4.9)	17.3 (4.2)	12.1 (3.4)	12.5 (3.4)
*R*_merge_ †	0.053 (0.191)	0.034 (0.058)	0.023 (0.051)	0.031 (0.066)	0.075 (0.385)	0.112 (0.518)	0.123 (0.506)	0.148 (0.634)
Wilson *B* factor (Å^2^)	12.10	11.34	16.40	10.80	20.53	74.49	14.57	67.70
Total observations	120868 (9130)	115163 (8727)	121574 (9127)	117979 (8726)	341635 (25171)	58058 (4220)	377399 (25186)	58939 (4294)
Total unique	32581 (2400)	31723 (2326)	32730 (2387)	31915 (2323)	30996 (2262)	5449 (389)	32092 (2318)	5845 (411)

† *R*_merge_(*I*) = 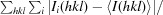 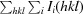.

## Footnotes

1 Supporting information has been deposited in the IUCr electronic archive (Reference: GM5033).
