# Supplementary material for: An evaluation of adhesive sample holders for advanced crystallographic experiments
Source: Acta Crystallogr D Biol Crystallogr. 2014 Aug 29;70(Pt 9):2390–400. doi: 10.1107/S1399004714014370 (PMC4157448; doi:10.1107/S1399004714014370)
Supplement: Supplementary file 4 [file d-70-02390-sup1.pdf]

Acta Crystallographica Section D

Volume 70 (2014)

Supporting information for article:

An evaluation of adhesive sample holders for advanced crystallographic experiments

Marco Mazzorana, Juan Sanchez-Weatherby, James Sandy, Carina M. C. Lobley and Thomas Sorensen

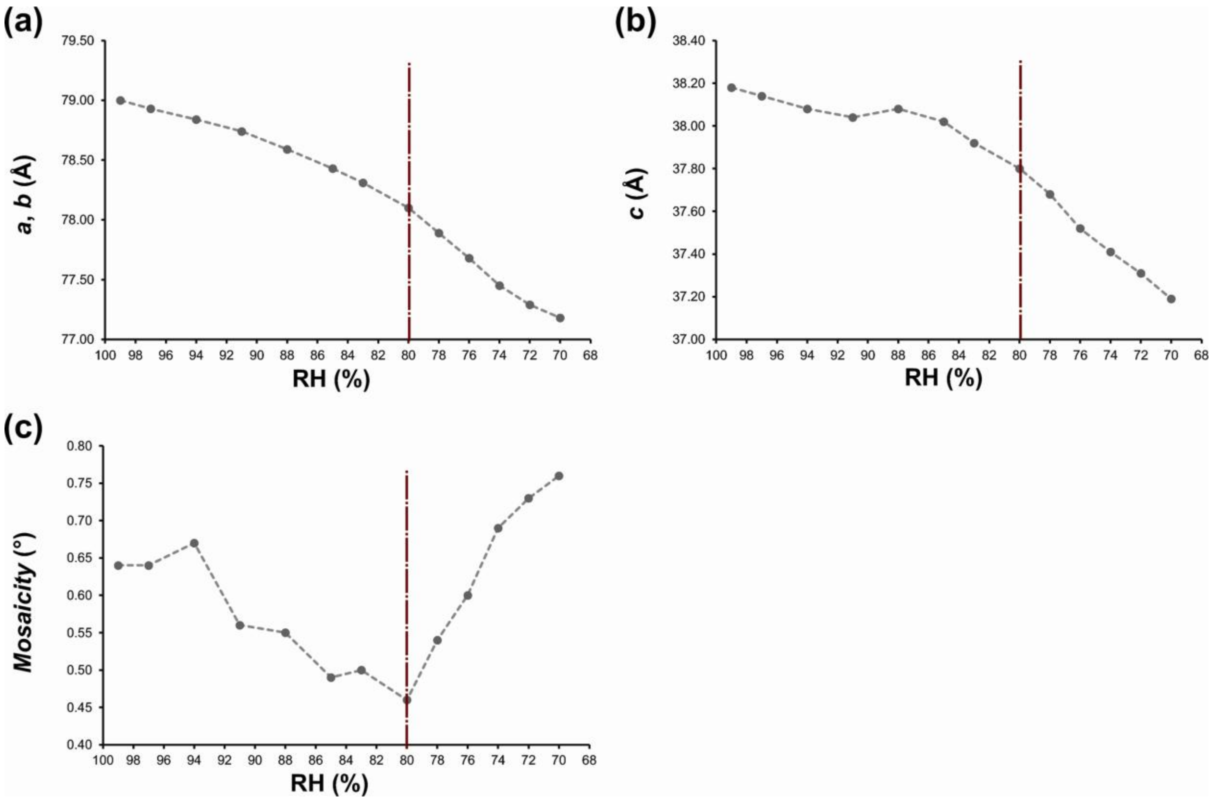

Figure S1 Dehydration experiment on a tetragonal lysozyme crystal. Plots showing changes of the *a* and *b* (a) and *c* (b) unit cell parameters as well as mosaic spread (c) as a function of decreasing relative humidity, for a tetragonal lysozyme crystal. Vertical lines indicate transition points for which the trend of one or more of the monitored parameters change as the RH changes.

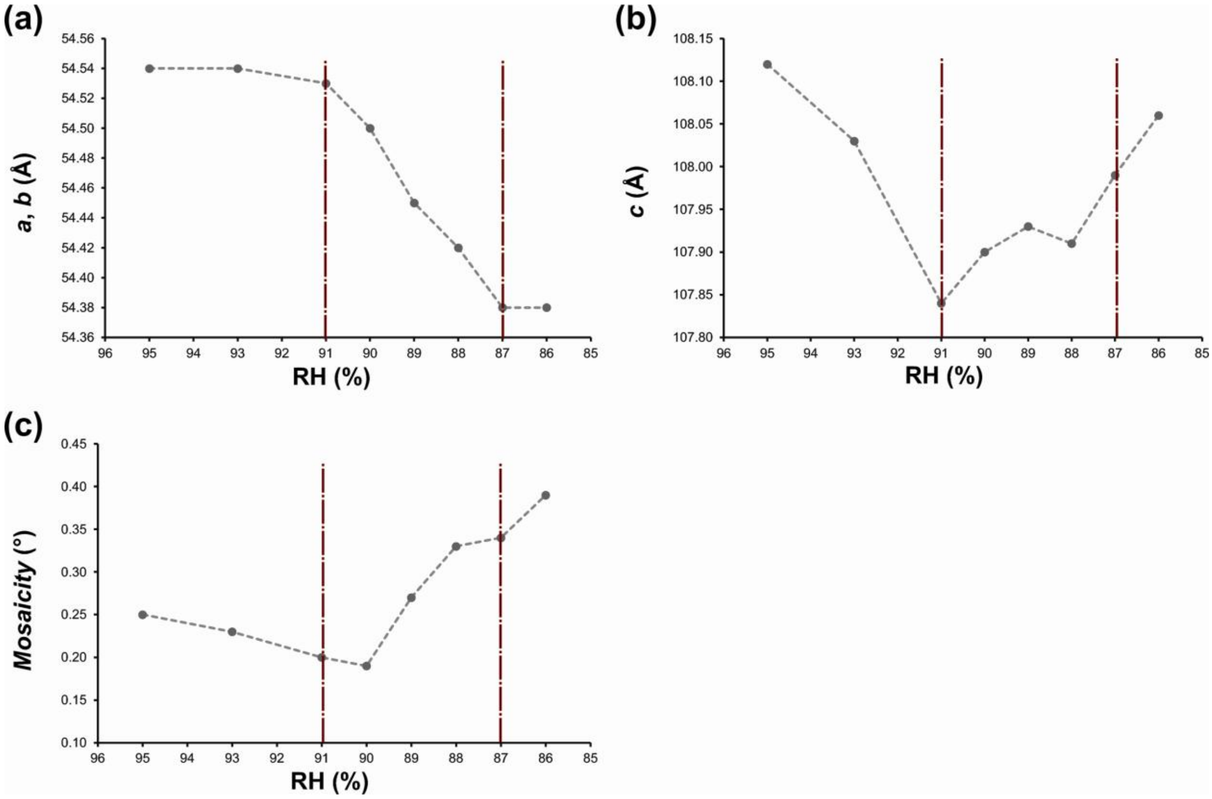

Figure S2 Dehydration experiment on a trigonal trypsin crystal. Plots showing changes of the *a* and *b* (a) and *c* (b) unit cell parameters as well as mosaic spread (c) as a function of decreasing relative humidity, for a trigonal trypsin crystal. Vertical lines indicate transition points for which the trend of one or more of the monitored parameters change as the RH changes.

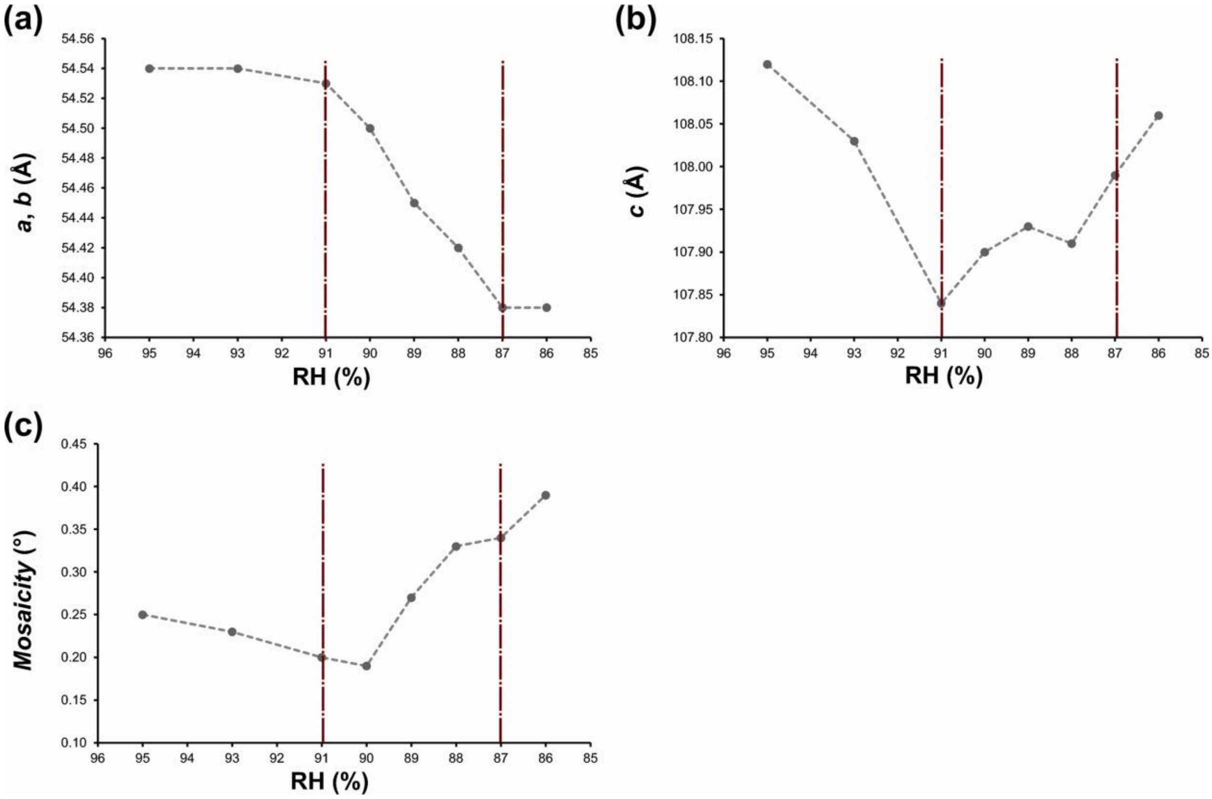

Figure S3 Dehydration experiment on a P4 DNA crystal. Plots showing changes of the *a* and *b* (a) and *c* (b) unit cell parameters as well as mosaic spread (c) as a function of decreasing relative humidity, for a tetragonal DNA crystal. Vertical lines indicate transition points for which the trend of one or more of the monitored parameters change as the RH changes.
